# Simple and Sensitive Stability-Indicating Ion Chromatography Method for the Determination of Cyclopropylamine in Nevirapine and Moxifloxacin Hydrochloride Drug Substances

**DOI:** 10.3797/scipharm.1107-14

**Published:** 2011-10-20

**Authors:** Pavan Kumar S. R. Kothapalli, Mukkanti Khagga, Nageswara Rao Mekala, John Prasanna Sigamani, Jagadeesh Kumar Vundavilli, Narendra Kumar Masani, Hemant Kumar Sharma

**Affiliations:** 1 Aurobindo Pharma Limited Research Centre, 313, Bachupally, Hyderabad-500 090, Andhra Pradesh, India; 2 J. N. T. University, Kukatpally, Hyderabad-500085, Andhra Pradesh, India

**Keywords:** Ion chromatography, Cyclopropylamine, Nevirapine, Moxifloxacin hydrochloride, Validation, Stress conditions

## Abstract

A simple and sensitive ion chromatography method has been developed for the determination of cyclopropylamine (CPA) in nevirapine (NEV) and moxifloxacin HCl (MOX) pharmaceutical drug substances. Efficient chromatographic separation was achieved on a Metrosep C4, 5 μm (250 mm × 4.0 mm) column. The mobile phase consists of 5 mM hydrochloric acid containing 10% (v/v) acetonitrile and was delivered in an isocratic mode at a flow rate of 0.9 mL min^−1^ at 27°C. A conductometric detector was used for the detection of the analyte. The drug substances were subjected to stress conditions including oxidation, thermal, photolytic and humidity for the evaluation of the stability-indicating nature of the method. The method was validated for specificity, precision, linearity, accuracy and solution stability. The limit of detection (LOD) and limit of quantification (LOQ) values are 0.10 μg mL^−1^ and 0.37 μg mL^−1^ respectively. The linearity range of the method is between 0.37 μg mL^−1^ and 1.5 μg mL^−1^ and the correlation coefficient is found to be 0.9971. The average recoveries of CPA in NEV and MOX are 97.0% and 98.0%, respectively.

## Introduction

Nevirapine (NEV) is an antiretroviral drug, specifically a non-nucleoside reverse transcriptase inhibitor. It is used to treat HIV, a retrovirus, with excellent biodistribution and good potential for use in combination with other antiretrovirals across the spectrum of HIV disease as well as in selected populations [1]. The chemical name of nevirapine is 11-cyclopropyl-4-methyl-5, 11-dihydro-6*H*-dipyrido[3,2-*b*:2′,3′-*e*][1,4]diazepin-6-one. The molecular formula is C_15_H_14_N_4_O and the molecular weight is 266.30.

Moxifloxacin is a new fourth-generation 8-methoxy-fluoroquinolone developed primarily for the treatment of community acquired pneumonia and upper respiratory tract infections. It is active against gram negative pathogens, gram positive cocci, aerobic intracellular bacteria, atypical organisms and anaerobic bacteria [2–3]. The chemical name of moxifloxacin hydrochloride (MOX) is 1-cyclopropyl-6-fluoro-8-methoxy-7-[(4a*S*,7a*S*)-octahydro-6*H*-pyrrolo[3,4-*b*]pyridin-6-yl]-4-oxo-1,4-dihydroquinoline-3-carboxylic acid hydrochloride. The molecular formula is C_21_H_24_FN_3_O_4_ · HCl and the molecular weight is 437.90. Cyclopropylamine (CPA) is used as raw material in synthetic process of NEV [4]. In this process, CPA is used to convert 2-chloro-*N*-(2-chloro-4-methylpyridin-3-yl)pyridine-3-carboxamide into *N*-(2-chloro-4-methylpyridin-3-yl)-2-(cyclopropylamino)pyridine-3-carboxamide, and also in synthetic process of MOX [5].

**Fig. 1:**
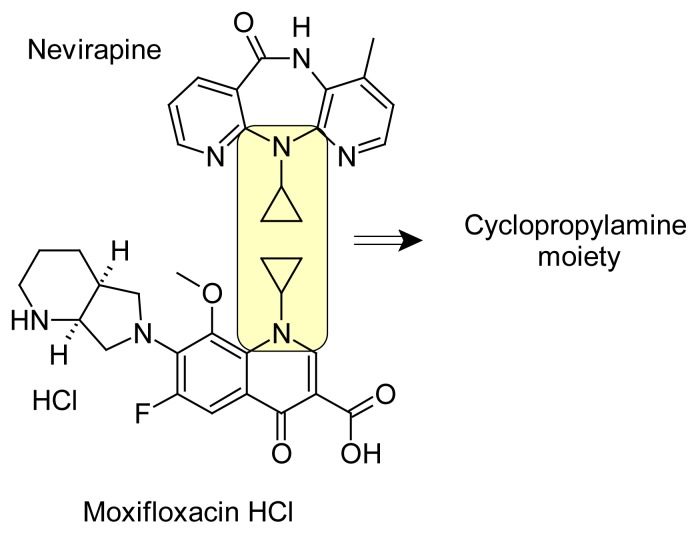
The CPA group in the chemical structures of NEV and MOX

CPA is a primary amine and has not been listed in residual solvents category in any of the regulatory guidelines and pharmacopoeias [6–8]. CPA has been widely applied as mechanistic probes of cytochrome P450 enzymes and other oxidative enzymes [9]. With regard to toxicity, its oral LD50 value in rat is 445 mg/kg [10]. Other than this there is no toxicity literature available to the best of our knowledge. The determination of CPA by gas chromatography (GC) [11] has been reported in literature. Additional references are available for the determination of volatile amines in air/gas samples by GC, free volatile amines after solid-phase microextraction by capillary GC, and ammonia and methylamines in natural waters by flow injection gas diffusion coupled with ion chromatography [12–14]. But, to the best of our knowledge, no literature is available for the determination of CPA in pharmaceutical drug substances by ion chromatography. According to International Conference on Harmonization (ICH) guidelines on impurities [15], any impurity other than an active moiety should be controlled with suitable limits in the drug substance irrespective of its harmful nature, and the limit of such impurities are not more than 0.1% [16]. This research paper describes the stability-indicating nature of the method by performing the forced degradation studies to drug substances as an active moiety of both the drug substances include CPA group. The chemical structures of NEV and MOX are depicted in Fig 1, wherein the CPA group is marked by encircling.

## Experimental

### Chemicals, reagents and samples

CPA standard, samples of NEV, MOX drug substances and their related substances [17, 18], ethyl nevirapine [Ph.Eur impurity A], descyclopropyl nevirapine [Ph.Eur impurity B], *n*-propylnevirapine [Ph.Eur impurity C], 6,8-difluoro [USP moxifloxacin related compound A or Ph.Eur impurity A], 6,8-dimethoxy [Ph.Eur impurity B], 8-ethoxy [Ph.Eur impurity C], 6-methoxy-8-fluoro [Ph.Eur impurity D] and 8-hydroxy [Ph.Eur impurity E] were gifted from APL Research Centre (A division of Aurobindo Pharma Ltd., Hyderabad, India). Analytical reagent (AR grade) hydrochloric acid, nitric acid, dipicolinic acid, sodium hydroxide, hydrogen peroxide, reagents, HPLC grade acetonitrile was purchased from E Merck, Mumbai, India. Highly purified water was obtained from millipore purification system (Millipore^®^, Milford, MA, USA). Photo stability studies were carried out in a photo stability chamber (Make: Sanyo, Model: PSC062.AHA.C, Sanyo Gallenkamp PLC, Leics, UK). Thermal studies were carried out in thermal oven (Make: Newtronic, Model: NW-CON-51, Mumbai, India) and humidity studies were carried out in humidity chamber (containing saturated aqueous solution of potassium nitrate by creating 90% relative humidity at 25°C).

### Ion chromatography

An ion chromatography system Metrohm 733 IC separation centre equipped with 732 conductometric detector, 709 IC pump, 762 IC interface and Metrohm 813 compact auto sampler with Metrohm IC net 2.3 data handling system (Metrohm, Switzerland) was used. The mobile phase was a 5 mM hydrochloric acid containing 10% acetonitrile by volume. The analysis was carried out on Metrosep C4, 5 μm, 250 mm long, 4.0 mm i.d. column at 27°C temperature. The mobile phase was delivered in an isocratic mode at a flow rate of 0.9 mL min^−1^. Detector scale range was 2 mS cm^−1^ and full scale was 10 μS cm^−1^. The thermostat temperature was set to 35°C and the conductivity flow cell constant was 16.3. The injection volume was 20 μL and the run time was 30 min. The retention time of the CPA peak is at about 5.2 minutes. The relative standard deviation for the peak areas of the six replicate injections for CPA peak was found to be less than 5.0%. This criterion has been used as system suitability criteria.

### Preparation of solutions

#### Standard solution

Accurately weigh and transfer 50 mg of CPA into a 100 mL volumetric flask containing 5 mL of mobile phase and make up to the volume with mobile phase. Dilute this solution to a final concentration of 0.75 μg/ml with mobile phase. Filter the solution through 0.45 μ porous membrane.

#### Sample solutions for NEV and MOX

Prepare a concentration of 1500 μg/ml of sample solutions with respective diluents individually and filter these solutions through 0.45 μ porous membrane.

## Results and discussion

### Method development and optimization

Based on column applications, for biogenic amines separation, Metrohm do suggest using C2 and C4 columns. Among these two columns, Metrosep C4 250 column is found to have excellent separation performance having a good column packing material (silica gel with carboxyl groups). Carboxyl groups in the stationary phase interact with analyte ions having opposite charge and lead to retain by the stationary phase. Therefore, the developmental work was started using this column. According to the application notes published by the Metrohm, mobile phase was prepared by mixing 1.7 mmol/L nitric acid and 0.7 mmol/L dipicolinic acid and using this mobile phase the CPA, NEV and MOX solutions were prepared and injected into the system. In these trials, CPA peak response was good. However, there was comparatively more noise, and remarkably the peak elution time of nevirapine was more than 60 min and the peak of moxifloxacin was not eluted. Therefore, the mobile phase was modified with 5 mmol hydrochloric acid instead of nitric acid without the addition of dipicolinic acid. In this trial, response of CPA was good and base line noise was low. However, the retention time of NEV peak was the same with regard to the previous trial. To improve the peak shape of CPA, acetonitrile was added as an organic modifier to mobile phase in various ratios, and finally with the ratio of 90:10% v/v, we achieved good peak shape of CPA as well as shorter elution time of NEV peak (at around 20 min). Then, the effect of concentration of hydrochloric acid was verified with 4 mmol, 6 mmol and 8 mmol, and there was no effect of peak shape and elution time. In this mobile phase, there was no interference observed from NEV and MOX related substances peaks and also with other inorganic cations like lithium, sodium, ammonium, potassium, magnesium, calcium and amines like monomethylamine, ethylamine, dimethylamine, *n*-propylamine, ethylmethylamine, diethyl amine and triethylamine. For MOX, mobile phase was chosen as diluent, while for NEV, because of poor solubility of drug substance in mobile phase, 1:1% v/v mobile phase and acetonitrile was chosen as diluent. Moreover, during method optimization, we performed forced degradation experiments for both drug substances, and it was found that the CPA peak is specific. The forced degradation experiment details have been reported in the method validation section.

### Method validation

The developed and optimized method was validated as per the ICH guidelines [19]**,** individually in terms of specificity, forced degradation studies (stability-indicating nature), sensitivity (limit of detection, limit of quantification), linearity, accuracy, precision and stability of sample solution.

#### Specificity

Specificity of the method was assessed by measuring the analyte response in the presence of all impurities related to drug substance. NEV and MOX related substances were spiked into sample solutions at pharmacopoeial levels [17, 18] along with and without CPA at about 500 μg/g in triplicate, and were injected into IC and determined the CPA content. The % difference between mean of CPA content in spiked individually and spiked with known related substances are 1.7 and 0.6 for NEV and MOX, respectively. The specificity experimental results are given in table 1.

Hence it was found that there was no interference observed in the determination of CPA content. Overlaid chromatograms of NEV spiked with all known related substances including CPA, NEV spiked with all known related substances excluding CPA, blank solution for NEV, MOX spiked with all known related substances including CPA, MOX spiked with all known related substances excluding CPA, blank solution for MOX with CPA standard solution are given in Fig 2(a) and in Fig 2(b) for NEV and MOX respectively.

Also, the blank solutions spiked with all known related substances of NEV and MOXwithout spiking CPA were injected and the interference was evaluated. Moreover, the stability-indicating nature of the method was verified through the forced degradation/stress studies of drug substances as the drug substance active moieties contain the CPA group.

In this study, NEV and MOX drug substances were subjected to the following stress conditions:

➢ Thermal stress: The drug substances were subjected to dry heat at 105°C for 120 h.
➢ Stress study under photolytic condition (as per ICH) [20]: A sample was exposed to photolytic degradation (white fluorescent light (10K Lux/120 h followed by UV light 200 watt-hours/m^2^.
➢ Stress study under humidity condition: A sample was exposed to degrade under 90% RH at 25°C for 120 h.
➢ Stress study under an oxidative condition: A sample solution was mixed with a 30% H_2_O_2_ solution and exposed to 85°C for 60 min.

The unstressed samples and each stressed sample were prepared to the required concentration and injected into IC using the analytical conditions. In unstressed samples, the result of CPA content was found to be not detected and in each stressed sample, there was no peak observed at the retention time of CPA. Hence, based on the above observation, the stressed conditions did not induce the degradation of API leading to CPA. Therefore, this method is specific, selective and having stability-indicating nature. We have applied the same methodology for pharmaceutical formulations like NEV tablets and MOX tablets. But, this method was not specific with respect to placebo interference experiment for NEV tablets. However, for MOX tablets, no placebo interference was observed.

#### Sensitivity (LOD and LOQ)

Sensitivity of method was determined by establishing the limit of detection (LOD) and limit of quantification (LOQ) values based on the residual standard deviation of a regression line and slope. CPA standard solution was injected into ion chromatograph from lower concentration (0.1 μg mL^−1^) to higher concentration (1.5 μg mL^−1^). A plot of peak area (mV*sec) versus concentration (μg mL^−1^) was drawn and LOD/LOQ values were predicted by STEYX (SD) and slope (S) method using the formula 3.3 × SD/S for LOD and 10 × SD/S for LOQ. The predicted concentration levels of LOD and LOQ solutions of CPA were verified for precision by analyzing six replicates. The achieved précised values are presented in table 2.

#### Linearity

The linearity of the method was determined by taking the same data obtained in LOD/LOQ prediction. The linearity of conductometric detector response of CPA at different concentrations was studied in the range of LOQ level i.e. 0.37 to 1.5 μg mL^−1^. The data was subjected to statistical analysis using a linear-regression model. The statistical parameters slope, intercept, STEYX and correlation coefficient values are calculated and shown in table 2.

#### Accuracy

Recovery of the method was carried out by spiking CPA to sample solutions in triplicate at three different levels at around 250, 500 and 750 μg/g, and the percentage recoveries were calculated. The average recovery values are reported in table 3.

#### Precision

The precision of the system was checked by injecting six replicates of CPA standard solution in different days and achieved RSD (%) was below 5.0. The repeatability (method precision) and reproducibility (ruggedness) of the method was studied by analyzing six sample solutions separately by spiking CPA at about 500 μg/g level. The ruggedness of the method was defined as the degree of reproducibility obtained by the analysis of the same samples (which is used in the Method precision) under a variety of conditions using different series of column, with different analysts on different days by preparing new standards and new mobile phase. Achieved results along with statistical data are given in table 4.

#### Solution stability

The sample solutions were prepared by the spiking CPA at about 500 μg/g level into drug substances. The stability of the solutions for both drug substances was tested by recording the chromatograms freshly prepared and at different intervals with the gap of every one hour up to 12 hours at 27°C temperature. The stability of solution was determined by comparing the area of the freshly prepared sample solutions with 12 hours stability sample solutions, and the % difference in area was found to be 1.7 and 2.2 for NEV and MOX respectively. The results indicated that the sample solution was stable for up to 12 hours at 27°C temperature.

#### Comparison with GC method

The LOD and LOQ values (0.10 & 0.37 μg mL^−1^) of the newly developed IC method were compared with the LOD and LOQ values (3 & 7 ppm) of GC method reported by P. Raghuram et al [11]. The results show that the present IC method is an alternative method for the determination of CPA and easy to handle with less cost of analysis.

## Conclusion

This stability-indicating ion chromatography method results demonstrated that the method is specific, sensitive having LOD and LOQ (0.10, 0.37 μg mL^−1^), linear (range: 0.37–1.5 μg mL^−1^), precise, rugged and accurate. The method is promising for the analysis of CPA in NEV and MOX drug substances because of its enhanced sensitivity and simple sample handling with low cost of analysis.

## Figures and Tables

**Fig. 2a:**
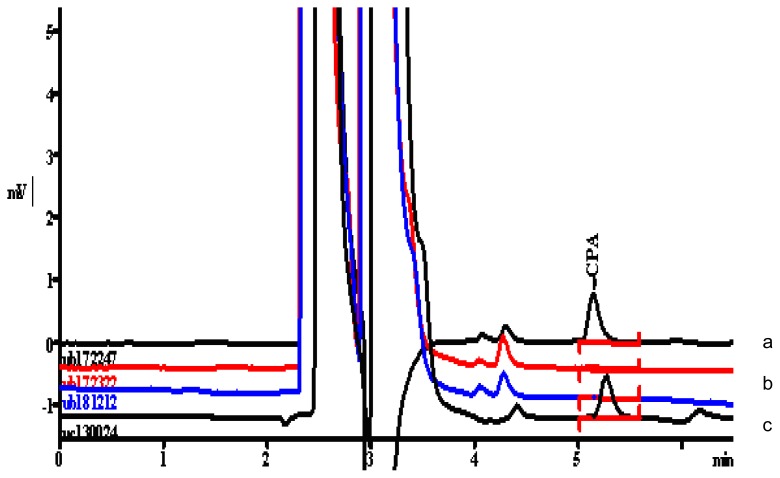
Overlaid chromatograms of (a) NEV spiked with all known related substances including CPA, (b) blank solution for NEV, (c) NEV spiked with all known related substances excluding CPA and (d) CPA standard solution.

**Fig. 2b:**
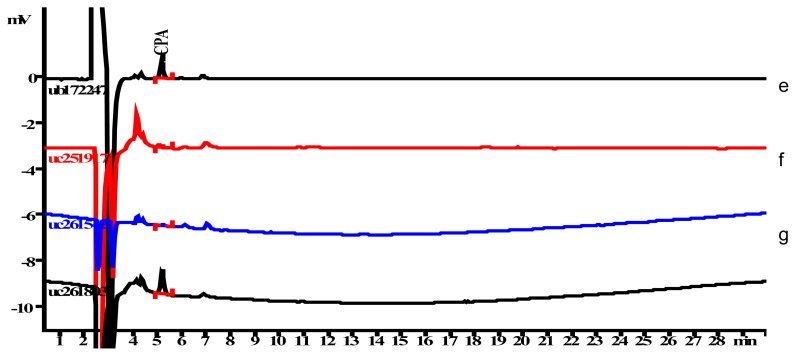
Overlaid chromatograms of (e) CPA standard solution, (f) blank solution for MOX (g) MOX spiked with all known related substances excluding CPA and (h) MOX spiked with all known related substances including CPA.

**Tab. 1:** Specificity of CPA

S.No	NEV		MOX	

	Without spiking of related substances	Spiked with related substances	Without spiking of related substances	Spiked with related substances

	CPA content (μg/g)		CPA content (μg/g)	

1	541	503	472	473
2	515	515	462	460
3	512	525	462	472
Average	523	514	465	468
SD	15.9	11.0	5.8	7.2
RSD (%)	3.0	2.1	1.2	1.5
% difference between spiked & unspiked	1.7		0.6	

**Tab. 2:** Statistical parameters obtained for CPA

Statistical parameters	Results
Correlation coefficient	0.9971
Concentration range (μg mL^−1^)	0.37–1.5
Intercept	−0.377
Slope (S)	9.207
STEYX (SD)	0.343
Limit of detection (μg mL^−1^)	0.10
Limit of quantification (μg mL^−1^)	0.37
Precision for Limit Of Detection RSD (%)	7.3
Precision for Limit Of Quantification RSD (%)	2.7

**Tab. 3:** Recovery (%) values for CPA

Accuracy (Average of 3 replicates)	Level-I	Level-II	Level-III	Overall recovery (%, Average of 9 replicates)
	**NEV**			
Added (μg/g)	249.3	499.0	749.0	97.0
Recovered (μg/g)	242.8	482.0	726.0	
Recovery (%)	97.4	96.6	96.9	
RSD (%)	0.9	1.3	1.6	

	**MOX**			

Added (μg/g)	252.7	505.0	757.0	98.0
Recovered (μg/g)	249.7	492.3	739.0	
Recovery (%)	98.8	97.5	97.6	
RSD (%)	3.5	1.2	0.5	

**Tab. 4:** Statistical data of precision obtained for determination of CPA

	NEV		

ID	Repeatability of standard^a^ (mV*sec)	Repeatability of sample^b^ (μg/g)	Reproducibility of sample^b^ (μg/g)
1	5.310	475	522
2	5.438	515	514
3	5.591	512	484
4	5.410	503	505
5	5.340	515	521
6	5.411	525	500
Mean	5.417	508	508
SD	0.098	17.41	14.49
RSD (%)	1.8	3.4	2.9
95% CI(±)	0.1	18.3	15.2

Overall statistical data (n=12)	Mean	508	
	SD	15.27	
	RSD (%)	3.0	
	95% CI(±)	9.7	

		**MOX**	

**ID**	**Repeatability of standard**^a^ (mV*sec)	**Repeatability of sample**^b^ **(μg/g)**	**Reproducibility of sample**^b^ **(μg/g)**

1	6.390	472	463
2	6.037	462	467
3	6.635	462	454
4	6.141	473	476
5	6.043	460	468
6	6.514	472	456
Mean	6.293	467	464
SD	0.255	6.08	8.17
RSD (%)	4.1	1.3	1.8
95% CI(±)	0.3	6.4	8.6

Overall statistical data (n=12)	Mean	465	
	SD	7.03	
	RSD (%)	1.5	
	95% CI(±)	4.5	

a …area of CPA;

b …content of CPA.
